# Requirements for Efficient Thiosulfate Oxidation in *Bradyrhizobium diazoefficiens*

**DOI:** 10.3390/genes8120390

**Published:** 2017-12-15

**Authors:** Sachiko Masuda, Hauke Hennecke, Hans-Martin Fischer

**Affiliations:** 1 RIKEN Center for Sustainable Resource Science, Yokohama 230-0045, Japan; 2 ETH Zurich, Institute of Microbiology, Vladimir-Prelog-Weg 4, CH-8093 Zurich, Switzerland hennecke@micro.biol.ethz.ch (H.H.); fischeha@ethz.ch (H.-M.F.)

**Keywords:** *Bradyrhizobium diazoefficiens*, thiosulfate, chemolithoautotroph, regulation, cytochrome

## Abstract

One of the many disparate lifestyles of *Bradyrhizobium diazoefficiens* is chemolithotrophic growth with thiosulfate as an electron donor for respiration. The employed carbon source may be CO_2_ (autotrophy) or an organic compound such as succinate (mixotrophy). Here, we discovered three new facets of this capacity: (i) When thiosulfate and succinate were consumed concomitantly in conditions of mixotrophy, even a high molar excess of succinate did not exert efficient catabolite repression over the use of thiosulfate. (ii) Using appropriate cytochrome mutants, we found that electrons derived from thiosulfate during chemolithoautotrophic growth are preferentially channeled via cytochrome *c*_550_ to the *aa*_3_-type heme-copper cytochrome oxidase. (iii) Three genetic regulators were identified to act at least partially in the expression control of genes for chemolithoautotrophic thiosulfate oxidation: RegR and CbbR as activators, and SoxR as a repressor.

## 1. Introduction

The α-proteobacterium *Bradyrhizobium diazoefficiens*, a facultative nitrogen fixer and root-nodule symbiont of soybean, is not only a versatile heterotroph but may also entertain a chemolithotrophic lifestyle in the non-symbiotic state. Hydrogen [1], carbon monoxide [2], and thiosulfate [3] have been reported to be the inorganic substrates that serve as electron donors for energy metabolism. In a previous study, Masuda et al. [3] demonstrated chemolithotrophic growth of *B. diazoefficiens* with thiosulfate as a reductant for oxic respiration according to the following equation: SSO_3_^2−^ + H_2_O + 2 O_2_ → 2 SO_4_^2−^ + 2 H^+^. The electrons derived from the oxidation of thiosulfate may also be used for the reduction of carbon dioxide as the sole carbon source (chemolithoautotrophy; [4]). CO_2_ can be replaced by an organic carbon source such as succinate, a growth phenotype that has been termed mixotrophy [3].

**Figure 1:**
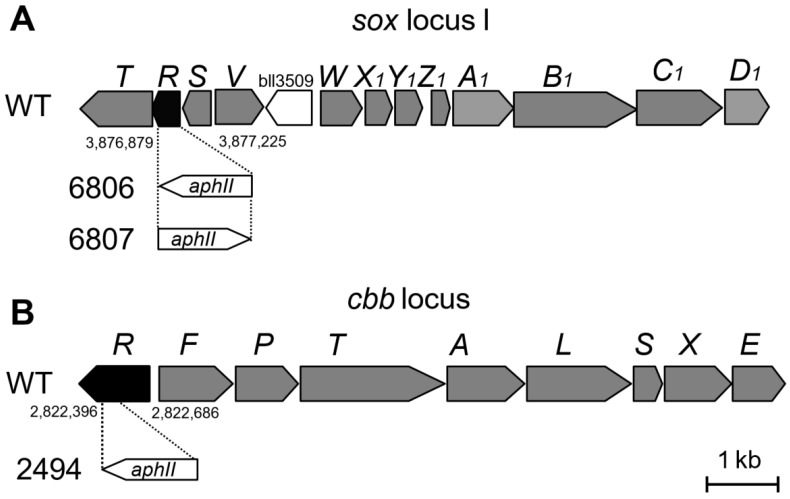
Genetic maps of the *sox* and *cbb* gene clusters in *B. diazoefficiens*. (**A**) Genes of *sox* locus I in the wild type, and the genotype of *soxR* deletion mutants 6806 (*∆soxR*, same orientation of the *aphII* gene) and 6807 (*∆soxR*, *aphII* in opposite orientation). The *∆soxR* deletion ends are defined by the nucleotide positions in the *B. diazoefficiens* genome. (**B**) Genes of the *cbb* cluster in the wild type, and the genotype of *cbbR* mutant 2494. The *∆cbbR* deletion ends are defined by the nucleotide positions in the genome.

An inspection of the *B. diazoefficiens* genome sequence [3,5] has revealed the existence of sulfur oxidation genes that typically occur in α-proteobacteria (*soxTRSVWXYZABCD*; Figure 1A) and code for proteins of the so-called Sox complex [6,7,8]. Therein, the SoxYZ proteins play a key role by binding thiosulfate covalently to a cysteine thiolate and forming a disulfide bridge in a reaction that releases the first couple of electrons: [SoxYZ]-S^−^ + SSO_3_^2−^ → [SoxYZ]-S-S-SO_3_^−^ + 2 e^−^ [8]. Curiously, the *B. diazoefficiens* genome harbors two *soxY* homologs (*soxY*_1_, *soxY*_2_; 3) of which, only *soxY*_1_ of *sox* locus I (Figure 1A) is essential for thiosulfate oxidation [3].

Following up on a previous publication [3], the present study aimed at addressing three unsolved aspects related to the physiology, biochemistry, and genetic regulation of thiosulfate oxidation. First, we wished to shed more light on the physiology of mixotrophic growth. To which extent are succinate and thiosulfate consumed concomitantly? Is succinate solely used as a carbon source or does it also replace thiosulfate as an energy source? Second, we wished to better understand the path electrons take from thiosulfate to oxygen as the terminal electron acceptor. The most prominent oxygen reductase during aerobic growth of *B. diazoefficiens* is the *aa*_3_-type cytochrome oxidase encoded by the *coxBAFC* genes [9]. Therefore, using appropriate *B. diazoefficiens* cytochrome mutants, we examined the possible involvement of a soluble *c*-type cytochrome and of *aa*_3_-type cytochrome oxidase in thiosulfate-dependent respiration. Third, we wished to investigate the genetic control of *sox* gene expression in *B. diazoefficiens*. Several indications as to which regulatory proteins might be involved came from studies with other bacteria. For example, negative control of *sox* gene expression had been discovered in *Paracoccus pantotrophus* GB17 and *Pseudaminobacter salicylatoxidans* KCT001 in which SoxR was shown to act as a repressor of *sox* genes in the absence of thiosulfate [10,11]. How *sox* gene induction occurs in the presence of thiosulfate is not known. The *sox* locus I of *B. diazoefficiens* (Figure 1A) harbors a *soxR*-homologous gene, which prompted us to explore its possible role in the regulation of thiosulfate oxidation. Another candidate regulator was the CbbR protein. Being an activator of the ribulose-1,5-bisphosphate carboxylase/oxygenase genes *cbbLS* and of genes for Calvin-Benson-Bassham-cycle enzymes in many autotrophic bacteria [12,13,14], CbbR might be a regulator also for chemolithoautotrophic growth of *B. diazoefficiens* with thiosulfate and CO_2_. A *cbbR*-like gene is present in the *cbb* cluster of the *B. diazoefficiens* genome ([4,5] Figure 1B). Finally, we were interested to know whether the two-component regulatory system RegSR [15,16] is involved in thiosulfate-dependent growth. The *B. diazoefficiens* RegS/RegR sensor/regulator system is a member of the highly conserved RegB/RegA family which is present in a wide range of bacteria and plays a fundamental role in the transcription of redox-regulated genes for numerous energy-generating and energy-utilizing processes such as photosynthesis, CO_2_ fixation, nitrogen fixation, hydrogen utilization, aerobic and anaerobic respiration, denitrification, and electron transport [17]. Therefore, thiosulfate oxidation might also be a target for control by RegSR in *B. diazoefficiens*.

## 2. Materials and Methods

### 2.1. Bacterial Strains, Media, and Growth Conditions

Bacterial strains and plasmids used in this study are listed in Table 1. Strain 110*spc*4 was used as the *B. diazoefficiens* wild type [18]. It is a spectinomycin-resistant derivative of USDA110 that, until recently, carried the species name *B. japonicum* [19]. *B. diazoefficiens* strains were pre-cultivated in peptone salts-yeast extract medium [18] supplemented with 0.1% L-arabinose. Precultures were washed twice with minimal medium lacking thiosulfate and succinate as described previously [3]. Then, fresh cultures were inoculated, and the strains were cultivated aerobically at 28 °C in minimal medium with sodium thiosulfate for chemolithoautotrophic growth, with sodium thiosulfate plus sodium succinate for mixotrophic growth, or with sodium succinate for heterotrophic growth, as described previously [3]. The concentrations of these ingredients will be mentioned in the context of the respective growth experiments. When appropriate, media for *B. diazoefficiens* cultivation contained antibiotics at the following concentrations (μg mL^−1^): spectinomycin, 100; streptomycin, 50; gentamycin, 100; kanamycin, 100; and tetracycline, 50 (solid medium) and 25 (liquid medium). *Escherichia coli* was grown in Luria-Bertani medium [20] containing the following concentrations of antibiotics for plasmid selection (μg mL^−1^): ampicillin, 200; kanamycin, 30; and tetracycline, 10.

### 2.2. Construction of soxR and cbbR Deletion Mutants

Plasmids for mutant construction and the resulting mutants are listed in Table 1. The *B. diazoefficiens* mutants were generated by marker replacement mutagenesis [29]. For deletion of *soxR*, upstream and downstream flanking regions of the *soxR* gene were amplified and cloned into pUC18, yielding pRJ6802. The *aphII* gene encoding kanamycin resistance was cut out from pBSL86 and inserted in either of two orientations at a KpnI site in-between the up- and downstream regions, resulting in pRJ6803 and pRJ6804. The mutated *soxR* DNA constructs were excised and cloned into the suicide plasmid pSUP202pol4, yielding pRJ6806 and pRJ6807. Mobilization of these plasmids into *B. diazoefficiens* 110*spc*4 was carried out via *E. coli* S17-1 and followed by screening for double recombination events, resulting in mutant strains 6806 (*∆soxR*, same orientation of the *aphII* gene) and 6807 (*∆soxR*, opposite orientation). The precise deletion ends with reference to the genome positions [5] are depicted in Figure 1A. A similar mutagenesis strategy was applied to generate a partial *cbbR* deletion mutant. To this end, a genomic 3,182-bp NotI-BamHI DNA fragment comprising *cbbR* and *cbbF’* was cloned in plasmid pRJ2492 from which it was excised as an EcoRI fragment and inserted in vector pSUP202pol4 to yield pRJ2493. A 290-bp *cbbR*-internal Acc65I-XhoI fragment was replaced by the kanamycin resistance cassette present on a 1,211-bp Acc65I-SalI fragment isolated from pBSL14. The resulting plasmid pRJ2494 was used for marker replacement mutagenesis to yield the *cbbR* mutant strain 2494. The precise deletion ends with reference to the genome positions [5] are shown in Figure 1B.

### 2.3. Expression of sox and cbb Genes

*B. diazoefficiens* strains were grown chemolithoautotrophically, mixotrophically, or heterotrophically in 100 mL of minimal medium (see above) in 500-mL Erlenmeyer flasks. Total RNA was extracted from chemolithoautotrophic cultures of the wild type and of mutants 6806 and 6807 at 6 days after inoculation, and of mutants 2494 and 2429 at 18 days after inoculation. Total RNA was extracted from mixotrophically and heterotrophically grown cultures after 30 h of growth. Cell harvest, RNA extraction, and cDNA synthesis were done as described previously [16]. The expression of *sox* and *cbb* genes was analyzed by quantitative reverse transcription-PCR (qRT-PCR) with techniques described previously [16], using suitable primers based on the respective *sox* and *cbb* gene sequences. The precise primer sequences are available from authors on request. For normalization we used the *sigA* gene which, based on our previous microarray data, was found to be unchanged under many different growth conditions (for example, see [16]).

### 2.4. Analysis of Thiosulfate, Succinate and Fumarate

Thiosulfate concentration was measured spectrophotometrically as previously described [3]. Extracellular succinate and fumarate concentrations were measured with a high-performance liquid chromatograph (Waters Alliance model 2690, Milford MA, USA) at 210 nm [30]. Two ml of cell cultures were harvested, filtered, and samples for HPLC analysis were taken from the supernatant. Sodium tartrate (10 mM) was added to the cell-free supernatant as an internal standard [30].

## 3. Results and Discussion

### 3.1. Concurrent Consumption of Thiosulfate and Succinate during Mixotrophic Growth

**Figure 2:**
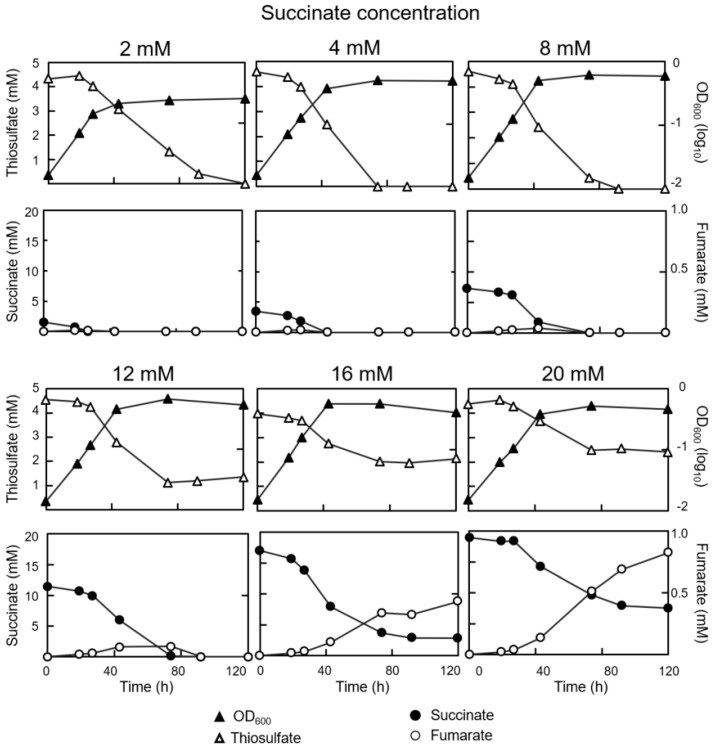
Mixotrophic growth of the *B. diazoefficiens* wild type. The growth media contained 4 mM thiosulfate and the amounts of succinate indicated above each pair of panels (from 2 to 20 mM). Cell growth was measured as optical density (OD) at A_600_, which is shown in the upper panels together with thiosulfate consumption. Succinate consumption and fumarate production are shown in the lower of panels. Two independent experiments were performed, and one of them is shown.

The performance of *B. diazoefficiens* growth in mixotrophic condition was explored. While the initial concentration of thiosulfate (4 mM) was kept constant, the initial concentration of succinate in the medium was varied between 2 mM and 20 mM (Figure 2; Table S1). Succinate at 2, 4, and 8 mM in the medium appeared to have a beneficial effect on growth because growth yields increased, and succinate was consumed completely without substantial accumulation of its immediate oxidation product, fumarate, in the medium. Surprisingly, thiosulfate was also consumed entirely in these cultures. Obviously, thiosulfate and succinate were consumed concomitantly if mixotrophic cultures contained 8 mM succinate or less. Even in the presence of higher succinate concentrations (12, 16, 20 mM) almost 50% of the available thiosulfate was still consumed while the remainder was detectable in the medium (Figure 2). Unphysiologically high succinate concentrations (16 and 20 mM) slightly impaired the final cell density, and neither succinate nor its oxidation product, fumarate was completely degraded by, or incorporated into, cells, which was indicated by the massive accumulation of unused fumarate in the medium. The most remarkable aspect of these results is that succinate exerts no or only partial catabolite repression over the use of thiosulfate. We interpret this to mean that succinate did either not or only partly replace thiosulfate as a source of reducing equivalents and hence, energy, but rather served predominantly as a carbon source. An alternative interpretation for the apparently obligate consumption of up to 50% thiosulfate in the presence of saturating amounts of succinate could be that thiosulfate was used as a sulfur source. This possibility appeared unlikely to us because the growth medium always contained a non-limiting amount of Mg-sulfate (3.25 mM) as the standard source of sulfur.

### 3.2. Chemolithoautotrophic Growth Partly Depends on Cytochrome c_550_ and the aa_3_-Type Cytochrome Oxidase

**Figure 3:**
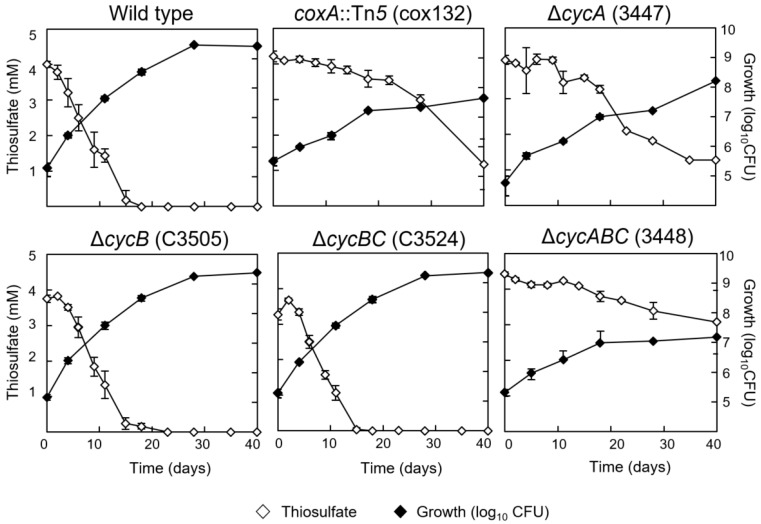
Contribution of cytochromes to chemolithoautotrophic growth of *B. diazoefficiens* with thiosulfate and CO_2_. The analyzed strains were the wild type and the cytochrome mutants cox132 (lacking subunit I of *aa*_3_-type cytochrome oxidase), 3447 (lacking cytochrome *c*_550_), C3505 (lacking cytochrome *c*_552_), C3524 (lacking cytochromes *c*_552_ and *c*_555_), and 3448 (lacking all three *c*-type cytochromes). The starting media contained 4 mM thiosulfate. The panels show thiosulfate consumption and growth measured as colony-forming units (CFU). CFU counts were assessed by taking samples from the cultures and plating out dilutions on agar plates with peptone-salts-yeast extract medium [18]. Shown are the mean values and standard deviation derived from three independent cultures.

Being a strictly oxic process, thiosulfate oxidation was expected to deliver electrons to the most prominent terminal oxygen reductase present in aerobically grown *B. diazoefficiens* cells, i.e., the *aa*_3_-type cytochrome oxidase [9]. To test this inference, a cytochrome *aa*_3_ subunit-I mutant (*coxA*::Tn*5*, strain cox132) completely defective in the activity of this oxidase was examined for chemolithoautotrophic growth with thiosulfate and CO_2_ and compared with the wild type. While all strains grew substantially slower under chemolithoautotrophic conditions compared with mixotrophic conditions, growth of the *coxA* mutant was particularly impaired, and thiosulfate consumption was strongly delayed in comparison with the wild type (Figure 3; Table S1). This suggested that the *aa*_3_-type cytochrome oxidase plays an important, though not essential role in thiosulfate oxidation. Since oxidases of this type receive electrons directly from reduced cytochrome *c*, we also tested which of the soluble *c*-type cytochromes previously identified in *B. diazoefficiens* [21,22,23] might fulfill such a task. Cytochromes *c*_552_ and *c*_555_, encoded by *cycB* and *cycC*, respectively, could be excluded because a *cycB* deletion mutant (strain C3505) and a *cycB*+*C* double deletion mutant (strain C3524) were proficient in chemolithoautotrophic growth and thiosulfate consumption just like the wild type (Figure 3). By contrast, a cytochrome *c*_550_ deletion mutant (Δ*cycA*, strain 3447) and a *cycA*+*B*+*C* triple mutant (strain 3448) displayed impaired chemolithoautotrophic growth and delayed thiosulfate consumption in a similar way as observed with the *coxA* mutant (Figure 3). It appears probable, therefore, that electrons derived from thiosulfate oxidation are transferred through cytochrome *c*_550_ to the *aa*_3_-type cytochrome oxidase for the reduction of O_2_. Notably, however, *cycA* and *coxA* mutants retained partial chemolithoautotrophic activity with thiosulfate, which suggests that alternative routes of electron transfer might exist in these mutants. Notably, *B. diazoefficiens* possesses two additional cytochrome oxidases and four additional ubiquinol oxidases that, collectively, might be responsible for the residual chemolithotrophic activity [9,31]. Whether one of these six oxidases plays an equally prominent role as the *aa*_3_-type oxidase in thiosulfate oxidation is difficult to predict, as we have no knowledge about the biochemical components (quinols, cytochromes) to which the enzymatic Sox complex of *B. diazoefficiens* delivers its electrons first.

### 3.3. Involvement of CbbR and RegR in the Regulation of Chemolithotrophic Thiosulfate Oxidation

**Figure 4:**
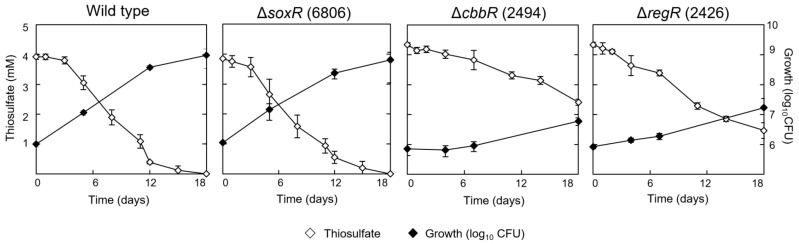
Contribution of transcription regulators to chemolithoautotrophic growth of *B. diazoefficiens* with thiosulfate and CO_2_. The analyzed strains were the wild type and the regulatory mutants 6806 (lacking the SoxR repressor), 2494 (lacking the CbbR activator), and 2426 (lacking the RegR response regulator of the two-component RegSR system). The starting media contained 4 mM thiosulfate. The panels show thiosulfate consumption and growth measured as colony-forming units (CFU). Shown are the mean values and standard deviation derived from three independent cultures.

Three regulatory proteins (SoxR, CbbR, RegR) were thought to be candidates for possibly controlling chemolithotrophic thiosulfate oxidation (see Introduction). To test their involvement in this phenotype, we constructed *soxR* and *cbbR* deletion mutants and analyzed chemolithotrophic thiosulfate oxidation in comparison with the wild type. The Δ*soxR* mutants were strains 6806 and 6807, and the Δ*cbbR* mutant was strain 2494 (Figure 1; Table 1). We included in this study a *regR* knock-out mutant (strain 2426) that was available from earlier work [15]. The two *∆soxR* strains showed a similar growth behavior as the wild type (shown in Figure 4 for strain 6806). Given the fact that SoxR is a repressor in other thiosulfate-oxidizing bacteria [10,11], the lack of a mutant phenotype in *B. diazoefficiens* Δ*soxR* strains did not come as a surprise. This made further analyses necessary in order to support a negative regulatory function of SoxR also in *B. diazoefficiens* (see below). CbbR and RegR are positive regulators [12,13,14,15,16]. Both proteins appeared to be involved in efficient thiosulfate-dependent chemolithoautotrophy of *B. diazoefficiens*, indicated by the increased generation time and decreased final cell density, and a diminished thiosulfate oxidation rate of the *cbbR* and *regR* mutants (Figure 4; Table S1). Since the mutations affected these properties only partly, we conclude that CbbR and RegR are important, but not essential activators involved in chemolithotrophic thiosulfate oxidation in *B. diazoefficiens*, which suggests the existence of other, so far unknown regulators in this process. While the importance of CbbR might be explained by its likely role as an activator of CO_2_ fixation genes, the role of RegR remains unknown. Gene expression studies were therefore designed to help clarify these issues.

### 3.4. Expression of Cbb and Sox Genes during Chemolithoautotrophic Growth

**Figure 5:**
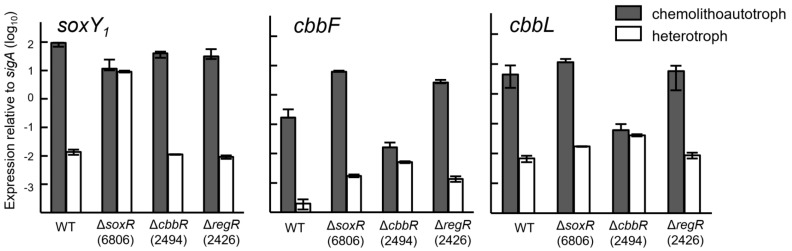
Expression of the *soxY*_1_, *cbbF*, and *cbbL* genes in the *B. diazoefficiens* wild type (WT) and in regulatory mutants *∆soxR* (6806), *∆cbbR* (2494) and *∆regR* (2426). Expression was measured by qRT-PCR in cells grown heterotrophically (with 4 mM succinate) and chemolithoautotrophically (with 4 mM thiosulfate). The expression of each gene under each condition had been normalized to that of the housekeeping sigma factor gene *sigA*. The column heights and error bars represent the means and standard deviations for three cultures where each culture was measured in triplicate.

**Figure 6:**
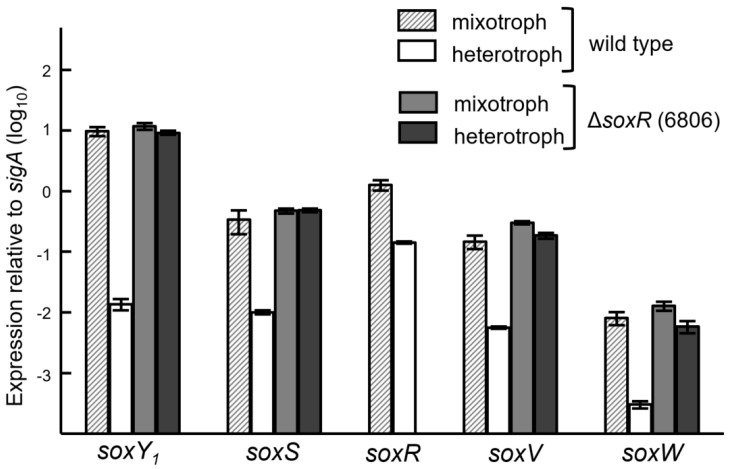
Expression of the *soxS*, *soxV*, *soxW*, and *soxY*_1_ genes in the *B. diazoefficiens* wild type and in the *soxR* mutant 6806. Expression was measured in cells grown under heterotrophic (4 mM succinate) and mixotrophic conditions (4 mM succinate plus 4 mM thiosulfate). The expression of each gene under each condition had been normalized to that of the housekeeping sigma factor gene *sigA*. The column heights and error bars represent the means and standard deviations of three cultures where each culture was measured in triplicate.

To support the physiologic observations with genetic data, the expression of the Calvin-cycle genes *cbbL* and *cbbF* and the thiosulfate oxidation gene *soxY*_1_ was examined. Using qRT-PCR, gene expression in cells grown chemolithoautotrophically was compared with that in heterotrophically grown cells. The strains used for this experiment were the wild type and the regulatory mutants Δ*soxR*, Δ*cbbR* and Δ*regR*. The results are displayed in Figure 5. A significant decrease in expression was observed only for the *cbbF* and *ccbL* genes in chemolithoautotrophically grown cells of the *cbbR* mutant (strain 2494). This confirms the role of CbbR as an activator for chemolithoautotrophy, especially CO_2_ fixation. By contrast, the growth defect caused by the *regR* mutation in strain 2426 (see Figure 4) was not reflected in a decreased expression of the investigated *soxY*_1_ and *cbbL* and *cbbF* genes (Figure 5). The chemolithoautotrophic growth defect of a *regR* mutant must therefore have other, so far unknown causes. The only effect seen in the *soxR* mutant (strain 6806) was an apparent de-repression of *soxY*_1_ expression in cells grown heterotrophically (Figure 5, and leftmost portion of Figure 6). This was the first indication in *B. diazoefficiens* that SoxR might be a negative regulator which, when mutated, leads to an enhanced expression of genes that are normally repressed in the wild type in the absence of thiosulfate.

### 3.5. Expression of the Sox Genes and Evidence for SoxR as a Negative Regulator

The expression studies in the *soxR* mutant background were expanded to include not only *soxY*_1_ but also additional genes of the *sox* gene cluster. Based on studies with *P. pantotrophus* GB17 and *P. salicylatoxidans* KCT001 [10,11], the *soxSR* and *soxVW* genes are divergently transcribed from SoxR-regulated promoters located between *soxS* and *soxV*. Gene expression was monitored with qRT-PCR under mixotrophic and heterotrophic growth conditions in the wild type and in the *soxR* mutant 6806 (Table 2, Figure 6). The *soxSR*, *soxV*, *soxW* and *soxY*_1_ genes were expressed most strongly in the wild type grown under mixotrophic condition where thiosulfate was present. In heterotrophic conditions, i.e., in the absence of thiosulfate, the *soxY*_1_ gene is poorly expressed in the wild type. The other genes also showed a decreased expression in heterotrophically grown cells, albeit less pronounced as compared with *soxY*_1_. In the *soxR* mutant strain 6806, however, the *soxS*, *soxV*, *soxW* and *soxY*_1_ genes showed an enhanced expression in heterotrophically grown cells as compared with respective wild-type cells. This is especially true for the *soxY*_1_ gene (leftmost portion of Figure 6). These results are fully in line with the purported role of SoxR as a repressor of *sox* genes in the absence of thiosulfate [10,11]. The data in Table 2 gives an estimate of the factors of de-repression of *sox* genes in the wild type when thiosulfate is present during mixotrophic growth. Again, the de-repression of *soxY*_1_ stands out most prominently. Biochemical transcription studies in vitro with purified components will now be necessary to understand the mechanism of SoxR-mediated negative control in the absence of thiosulfate.

In conclusion, our study contributes to a better understanding of the complex and versatile physiology of *B. diazoefficeins*. While studying mixotrophic and chemolithoautotrophic growth of *B. diazoefficiens* wild type and a series of defined mutants, we have identified two members of the electron transfer pathway from thiosulfate to oxygen, cytochrome CycA and *aa*_3_-type cytochrome oxidase CoxBAFC. Moreover, we have demonstrated for the first time the involvement of CbbR and RegR in autotrophic growth of *B. diazoefficiens*, which expands the functional relevance of these regulatory proteins in complex genetic networks present in this bacterium. At this stage, the molecular signals which are sensed and transduced by CbbR and RegR remain to be identified. The same applies to the previously uncharacterized SoxR protein for which we demonstrated a role in negative regulation of *sox* genes in heterotrophically grown cells of *B. diazoefficiens*.

## Figures and Tables

**Table 1:** Bacterial strains and plasmids used in this study.

Strain or Plasmid	Relevant Genotype or Phenotype	Reference or Source
Strains		
*Bradyrhizobium diazoefficiens*		
110*spc*4	Sp^r^ wild type	[18]
6806	Sp^r^ Km^r^ *soxR*::*aphII* (same orientation)	This study
6807	Sp^r^ Km^r^ *soxR*::*aphII* (opposite orientation)	This study
2494	Sp^r^ Km^r^ *cbbR*::*aphII*	This study
2426	Sp^r^ Sm^r^ *regR*::Ω	[15]
cox132	Sp^r^ Km^r^ *coxA*::Tn*5*	[21]
3447	Sp^r^ Gm^r^ *cycA*::*gen*	[22]
C3505	Sp^r^ Sm^r^ *cycB*::Ω	[23]
C3524	Sp^r^ Sm^r^ Km^r^ *cycB*::Ω *cycC::aphII*	[22]
3448	Sp^r^ Sm^r^ Km^r^ Gm^r^ *cycA*::*gen cycB*::Ω *cycC::aphII*	[22]
*Escherichia coli*		
BL21 (DE3)	*E. coli* B F^−^ *dcm ompT hsdS*_B_ (r_B_^−^ m_B_^−^) *gal* λ(DE3)	[24]
DH5α	*supE44* ∆*lacU169* (Φ80*lacZ*∆M15) *hsdR17 recA1 gyrA96 thi-1 relA1*	BRL *^a^*
S17-1	Sm^r^ Sp^r^ *hsdR* (RP4-2 *kan*::Tn*7 tet*::Mu; integrated in the chromosome)	[25]
Plasmids		
pBSL14	Ap^r^ Km^r^	[26]
pBSL86	Ap^r^ Km^r^	[26]
pSUP202pol4	Tc^r^ (pSUP202) *oriT* of RP4	[27]
pUC18	Ap^r^ cloning vector	[28]
pBluescript II SK+	Ap^r^ cloning vector	Stratagene *^b^*
pRJ2492	Ap^r^ (pBluescript II SK+) genomic 3,182-bp NotI-BamHI fragment spanning *cbbR*-*cbbF’*	This study
pRJ2493	Tc^r^ (pSUP202pol4) *cbbR*-*cbbF’*, 2,353-bp EcoRI fragment of pRJ2492	This study
pRJ2494	Tc^r^ Km^r^ (pSUP202pol4) *∆cbbR*::*aphII* (same orientation), 1,211-bp Acc65I-SalI fragment of pBSL14 inserted into Acc65I-XhoI-digested pRJ2493	This study
pRJ6802	Ap^r^ (pUC18) overlapped-extension PCR-generated in frame deletion of *soxR* on 1.6-kb EcoRI-HindIII fragment cloned into pUC18	This study
pRJ6803	Ap^r^ Km^r^ (pRJ6802) *∆soxR*::*aphII*, 1,260-bp KpnI fragment from pBSL86 inserted into KpnI site of EcoRI-HindIII fragment (same orientation as *soxR*)	This study
pRJ6804	Ap^r^ Km^r^ (pRJ6802) *∆soxR*::*aphII*, 1,260-bp KpnI fragment from pBSL86 inserted into KpnI site of EcoRI-HindIII fragment (opposite orientation to *soxR*)	This study
pRJ6806	Km^r^ Tc^r^ (pSUP202pol4) *∆soxR*::*aphII*, 2,894-bp EcoRI/HindIII fragment from pRJ6803 inserted into SmaI site (*aphII* and *soxR* in same orientation)	This study
pRJ6807	Km^r^ Tc^r^ (pSUP202pol4) *∆soxR*::*aphII*, 2,894-bp EcoRI/HindIII fragment from pRJ6804 inserted into SmaI site (*aphII* and *soxR* in opposite orientation)	This study

Sp^r^, spectinomycin resistant; Km^r^, kanamycin resistant; Sm^r^, streptomycin resistant; Gm^r^, gentamicin resistant; Ap^r^, ampicillin resistant; Tc^r^, tetracycline resistant. *^a^* Gaithersburg MD, USA; *^b^* La Jolla CA, USA.

**Table 2:** Ratio of relative expression of *sox* genes in *B. diazoefficiens* wild type and *soxR* mutant 6806 grown under mixotrophic and heterotrophic conditions ^a^.

Genes	Wild Type	6806 (Δ*soxR*)
*soxR*	16 ± 8	---
*soxS*	50 ± 17	1.2 ± 0.5
*soxV*	50 ± 26	0.7 ± 0.3
*soxW*	90 ± 45	0.8 ± 0.7
*soxY*_1_	4500 ± 1500	1.4 ± 0.4

^a^ Expression of each gene under heterotrophic and mixotrophic growth condition was normalized to that of the housekeeping sigma factor gene *sigA*. Numbers reflect the relative expression level in cells grown under mixotrophic condition divided by the expression levels in cells grown under heterotrophic conditions. The means ± standard deviations of three biological replicates are shown.

## Supplementary Materials

The following are available online at www.mdpi.com/2073-4425/8/12/390/s1. Table S1: Generation time of all strains used in this study grown under the indicated conditions.
