# Supplementary material for: Requirements for Efficient Thiosulfate Oxidation in Bradyrhizobium diazoefficiens
Source: Genes (Basel). 2017 Dec 15;8(12):390. doi: 10.3390/genes8120390 (PMC5748708; doi:10.3390/genes8120390)
Supplement: Supplementary file 1 [file genes-08-00390-s001.pdf]

Table S1: Generation time of all strains used in this study grown under the indicated conditions

Strain	Genotype	Generation time [a]						
		Mixotrophic growth (hours)						Chemolithoautotrophic growth (days)
		2 mM	4 mM	8 mM	12 mM	16 mM	20 mM	
110spc4	Wild type	7.7	8.9	8.1	8.1	7.3	8.5	1.4
cox132	*coxA*::Tn*5*	-	8.7	-	-	-	-	3.1
3447	Δ*cycA*	-	9.2	-	-	-	-	1.7
C3505	Δ*cycB*	-	-	-	-	-	-	1.2
C3524	Δ*cycBC*	-	-	-	-	-	-	1.5
3448	Δ*cycABC*	-	7.9	-	-	-	-	2.3
6806	Δ*soxR*	-	8.7	-	-	-	-	1.4
2494	Δ*cbbR*	-	7.5	-	-	-	-	4.0
2426	Δ*regR*	-	8.2	-	-	-	-	3.5

[a] Generation times were calculated from the respective growth curves shown in Fig. 2, 3, 4.
